# Echocardiographic Advances in Dilated Cardiomyopathy

**DOI:** 10.3390/jcm10235518

**Published:** 2021-11-25

**Authors:** Andrea Faggiano, Carlo Avallone, Domitilla Gentile, Giovanni Provenzale, Filippo Toriello, Marco Merlo, Gianfranco Sinagra, Stefano Carugo

**Affiliations:** 1 Cardiology Unit, Internal Medicine Department, Fondazione IRCCS Ca’ Granda Ospedale Maggiore Policlinico, University of Milan, 20122 Milan, Italy; carloavallone95@gmail.com (C.A.); domitilla.gentile@gmail.com (D.G.); giovanniprovenzale91@gmail.com (G.P.); filippo.toriello@gmail.com (F.T.); stefano.carugo@unimi.it (S.C.); 2 Cardiothoracovascular and Medical Surgical and Health Science Department, Azienda Sanitaria Universitaria Giuliano Isontina (ASUGI), University of Trieste, 34149 Trieste, Italy; marco.merlo79@gmail.com (M.M.); gianfranco.sinagra@asugi.sanita.fvg.it (G.S.)

**Keywords:** dilated cardiomyopathy, echocardiography, echocardiographic advances, global longitudinal strain, artificial intelligence, heart failure, ejection fraction, left ventricular remodeling

## Abstract

Although the overall survival of patients with dilated cardiomyopathy (DCM) has improved significantly in the last decades, a non-negligible proportion of DCM patients still shows an unfavorable prognosis. DCM patients not only need imaging techniques that are effective in diagnosis, but also suitable for long-term follow-up with frequent re-evaluations. The exponential growth of echocardiography’s technology and performance in recent years has resulted in improved diagnostic accuracy, stratification, management and follow-up of patients with DCM. This review summarizes some new developments in echocardiography and their promising applications in DCM. Although nowadays cardiac magnetic resonance (CMR) remains the gold standard technique in DCM, the echocardiographic advances and novelties proposed in the manuscript, if properly integrated into clinical practice, could bring echocardiography closer to CMR in terms of accuracy and may certify ultrasound as the technique of choice in the follow-up of DCM patients. The application in DCM patients of novel echocardiographic techniques represents an interesting emergent research area for scholars in the near future.

## 1. Introduction

The current definition of dilated cardiomyopathy (DCM) could appear relatively simple; namely, a heart muscle disease characterized by left ventricular (LV) or biventricular dilation and systolic dysfunction in the absence of pressure or volume overloads or coronary artery disease sufficient to explain the dysfunction [1,2]. Actually, DCM is being used as an ‘umbrella’ term describing the common pathway of different diseases and gene—environment interactions [3,4]. Despite the overall survival of patients with DCM has improved significantly in the last decades [5], a non-negligible proportion of DCM patients still shows an unfavorable prognosis [6].

Echocardiography is crucial in the diagnosis, stratification, management and follow-up of patients with DCM [7,8]. Nearly 70 years have passed since Inge Edler and Hellmuth Hertz began using M-mode echocardiography as a diagnostic tool for cardiovascular disease in 1953 [9]. Ever since, echocardiography has evolved from a simple M-mode imaging technique to an extensive array of advanced technologies [10]. Moore’s Law predicts that every two years the performances of technology will double while their cost will fall by half [11]. Several promising echocardiography techniques, including artificial intelligence (AI) applications, are now available for clinicians [12]. Although the implementation of these echocardiographic advances in daily clinical practice can be of great help in the diagnosis and management of DCM, their diffusion is currently still very limited. For this reason, this review aims to offer a brief overview of some new developments in echocardiography and their promising applications in DCM. The manuscript is structured in mini-sections that delve into each specific area of a comprehensive transthoracic echocardiographic examination.

## 2. Left Ventricular Dimensions, Geometry and Systolic Function 

Echocardiographic DCM diagnostic criteria traditionally consisted of the presence of LV ejection fraction (EF) <45% and/or fractional shortening <25% and left ventricular end diastolic dimension (LVEDD) >112% of predicted value corrected for age and body surface area (BSA) [13]. Actually, the 2016 European Society of Cardiology (ESC) position paper revised the definition of DCM and established that detecting a non-ischemic Left ventricular ejection fraction (LVEF) <50% is sufficient to diagnose DCM [14], a clinical entity that could include also non-dilated and arrhythmogenic forms.

The EF and fractional shortening are part of the standard echocardiographic parameters used to assess LV dimensions, geometry and systolic function, among which there are also: dP/dT and cardiac output (all load dependent), tissue doppler mitral annulus velocity and speckle tracking strain (less load dependent). EF is defined as stroke volume (SV) indexed to EDV and is a pillar of clinical practice, even if is deeply influenced by preload, afterload, heart rate and ventricular geometry. The biplane Simpson’s rule is generally recommended to calculate EF, although there are significant limitations: suboptimal endocardial definition in up to 15% of patients, poor reproducibility of measurements and inability to reflect regional LV function. A better description of LV mechanics derives from a non-invasive evaluation of myocardial deformation, namely myocardial strain [15].

Global longitudinal strain (GLS) is a dimensionless variable and represents the percent change in myocardial length between two points over the cardiac cycle. The measurements of myocardial segments’ length change are performed by tracking the shift of myocardial speckles via an algorithm [16]. Since the measures are done directly, the strain is less dependent on ventricular loading conditions, compliance of myocardium and geometrical components [17]. LV deformation through quantification of strain could be made by cardiac magnetic resonance (CMR) tissue tagging or feature tracking approaches or more frequent by tissue Doppler or speckle tracking echocardiography (STE). Tissue displacement is used to calculate myocardial deformation, a parameter shown to provide unique information on regional and global ventricular function, highly reducing inter and intra-observer variability [18]. Additionally, GLS is a more powerful predictor of prognosis and outcomes in patients with heart failure (HF) with reduced ejection fraction (HFrEF) and DCM [19,20] (both ischemic and non-ischemic) and represents a novel parameter for a better selection of patients for implantable cardiac defibrillator (ICD) implantation as a more accurate predictor of ventricular arrhythmias [21]. LV GLS correlates well with invasive hemodynamic parameters [22,23] and independently predict LV reversed remodeling (discussed further below) in patients with DCM: patients with higher baseline GLS presented better LV functional recovery leading to less adverse clinical events [24].

The great improvement in DCM prognosis in the last decades [25] seems to be related to the left ventricular reverse remodeling (LVRR): the decrease in dimensions and the normalization of LV shape associated with a significant improvement of pump function. The importance of an accurate initial diagnosis goes hand in hand with continuous, individualized follow-up with quantitative echocardiographic assessment. Since patients affected by non-ischemic DCM who demonstrate left ventricular contractile reserve have lower mortality, cardiac events/hospitalizations [26] and more frequent LVRR [27], the individualized-evaluation of contractile reserve with stress-echocardiography offers an important prognostic indicator in the management of these patients.

**Figure 1:**
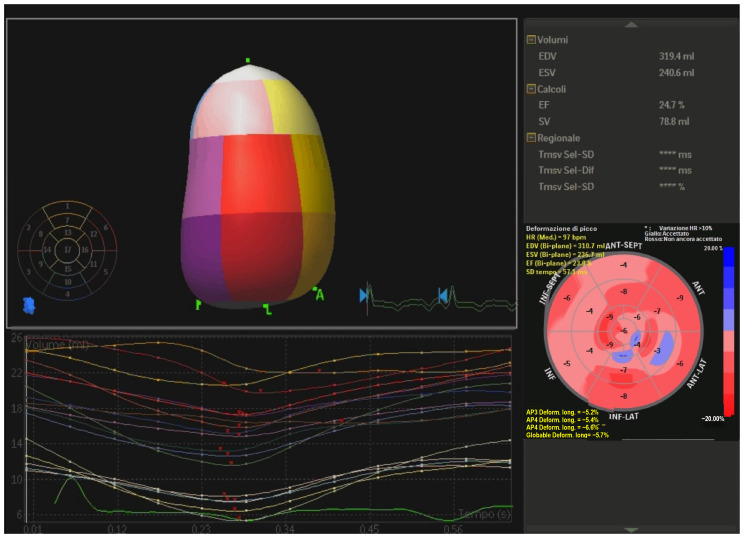
LVEF obtained with 3D echocardiography and GLS measurements in a patient with DCM. In the 3D reconstruction different colors indicates different LV segment. In the Bull’s eye display of GLS intense shades of red indicate optimal GLS values (−20%), while less intense shades, white color and different intensities of blue indicate sub-optimal and pathological GLS values (+20%). EDV = end-diastolic volume; ESV = end-systolic volume; EF = ejection fraction; SV = stroke volume, HR = heart rate, GLS = global longitudinal strain; DCM = dilated cardiomyopathies.

More recent refinements in LV echocardiographic characterization could be found in the following echo parameters and techniques:

- Three-dimensional (3D) echocardiography. This technique improves reproducibility of LV volumes with an accuracy similar to CMR [28]. It is an ideal tool for measuring LV volumes because no geometric assumptions about shape are needed and is unaffected by foreshortening of the apex. Conversely, it is characterized by lower temporal resolution, high dependance on image quality and there are only a few data available on reference values. Real-time 3D echocardiographic systems have been developed [29,30,31]; these systems utilize fully samples matrix array transducers capable of acquiring volumetric data. 3D echocardiography (Figure 1) is more reproducible and superior to 2D in the accuracy of LV volumes and EF measurements [32], however, despite clear recommendations in the guidelines, its use is still limited probably due to the long learning curve [33]. Moreover, 3D-STE has been proven a reliable tool for the evaluation of LV systolic function in patients with non-ischemic DCM with a good interobserver, intraobserver and test-retest reliability [34] and a good correlation with data obtained from CMR [35].
- Non-invasive left ventricular pressure-strain loop (PSL) and global work index (GWI). Myocardial work (MW) is a new parameter that considers both myocardial deformation and after-load. It is calculated combining LV strain and non-invasively estimated LV pressure curves. The area within the PSL represent an index of MW, and the following parameters can be determined [36]: GWI, as the total work within the PSL area from mitral closure to opening; constructive MW, as the work performed by LV responsible for LV systolic ejection; wasted MW work, as work that does not contribute to ejection; MW efficiency, as constructive MW/constructive MW plus wasted MW. These equations permit to better understand the relationships between LV remodeling and increased after-load under different loading conditions. Moreover GWI and constructive work (GCW) are powerful and independent predictors of outcome in patients with DCM and advanced HFrEF [37]. GCW better predicts LV fibrosis than GLS, and could represent a surrogate marker for detection of fibrosis in addition to CMR [38].
- Reverse remodeling index. Although DCM is classically defined as above, conventional geometric parameters used have not been demonstrated to have a prognostic value [39,40,41,42]. Remodeling index is a novel geometric criterion calculated as the cubic root of LVEDV divided by mean LV wall thickness. It was shown to be an equivalent in evaluating ventricular maladaptive remodeling [43] in hypertensive patients. Xu et al. recently investigated the value of this new marker in DCM cohorts as an independent predictor of all-cause mortality, heart transplantation and HFrEF readmissions [44].
- Post-systolic shortening (PSS) and early systolic lengthening (ESL). Objective measures of cardiac function are supplied by PSS and ESL [45], both parameters reflecting the paradoxical deformation of the myocardium. During systolic ejection approximately one third of segments display physiologic PSS and the same applies to ESL [46,47]. These indices are quantified by invasive and non-invasive methods, like strain-rate, tissue Doppler imaging, speckle tracking. Pathological deformation typically occurs during acute ischemia, and it is a predictor of recovery and ischemic memory, and, recently, the ability of PSS and ESL to predict adverse cardiac outcomes has been demonstrated in a wide range of special population [48,49,50,51,52].
- Mitral valve complex (MVC) tissue longitudinal elongation. It is clear that LV sphericity is associated with impaired LVEF, functional mitral regurgitation (MR) and poor prognosis [53,54] in DCM. LV shape becomes spherical and myocardial tissue elongates transversally [55]. The dynamic nature of MVC is quite different from myocardium [56] and so it may not be affected while myocardium spherically remodels. Hence, MVC may potentially deforms regionally and not uniformly leading to limited elongation of LV base and asymmetrically less elongation of the MVC. LV sphericity can be calculated via 2D echocardiography by the ratio of transverse cavity dimension to the longitudinal diameter at end diastole, so a higher sphericity index represents greater LV sphericity: therefore, remodeling is predominant at bases and not as clear at the apex leading to new promising diagnostic tools and interventions [57].
- Artificial intelligence and machine learning (ML). AI is historically defined as the ability of computer systems to perform tasks that would usually require human levels of intelligence [58]. AI has recently become a research hotspot in echocardiography [59], although this is yet not so advanced and in 1978 was already used to estimate the waveform of anterior mitral leaflet via M-mode. The progress of new technologies, such as deep learning and neural networks, has dramatically boosted the strength of echocardiography. Advantages of using ML models includes automated analysis, reducing observer variability, providing more consistent and reproducible data, allowing big data analysis, predicting future data, helping in therapeutic decision making, and nevertheless it is time and cost saving, reducing unnecessary further investigations [60]. Specifically, LV evaluation can be derived from algorithms built mimicking what a trained sonographer can do [61,62]. The same applies to GLS analysis: AI technique can help recognize standard views, perform timing of cardiac events, trace the myocardium, achieve motion estimation and measure GLS in <15 s [63]. In the context of DCM, a vector machine classifier was addressed to study the ventricular wall changes to discriminate between normal and dilated pattern [64]. The inclusion of ML models in echocardiography appears very promising, as they are able to precisely identify various echocardiographic features and predict outcomes, without the limitations related to human interpretation: a novel multicenter research shown that AI-based LV analysis were predictor of mortality [65]. There are still doubts about insufficient standardization of echocardiography, poor robustness and scant generalization of the models in clinical application. The continue advancement of AI technology will gradually remodel the forthcoming of echocardiography to grant practical auxiliary assistance for cardiologists [66].

Table 1 shows a comparison between Standard and Emerging echocardiographic techniques to assess left ventricular dimensions, geometry and systolic function in dilated cardiomyopathy.

## 3. Left Ventricular Diastolic Function 

LV diastolic function plays an important role in determining LV filling and stroke volume in DCM. Diastolic dysfunction is associated with worse outcomes, including total mortality and hospitalizations due to HF [67,68]. The gold standard for the evaluation of diastolic dysfunction, broadly defined as impaired LV relaxation and increased myocardial stiffness, is invasive left heart catheterization [69]. However, echocardiography, due to the versatility and the noninvasive nature, is today the preferred method to assess LV diastolic function. The echocardiographic assessment of diastolic function, together with left/right ventricular involvement, left atrial (LA) enlargement and mitral regurgitation, improves the prognostic stratification in DCM and helps predict LVRR [2]. The current American Society of Echocardiography/European Association of Cardiovascular Imaging 2016 guidelines are based on two assumptions: (1) none of the indices should be used in isolation; (2) the diastolic function evaluation of patients with reduced LVEF follows a different approach [70]. The guidelines suggest the application of two algorithms, one to screen for the presence of diastolic dysfunction and the other to grade diastolic dysfunction if it is found to exist. The first algorithm relies on four parameters: annular e’ velocity (septal e’ < 7cm/s, lateral e’ <10 cm/s), average E/e’ ratio >14, LA maximum volume index >34 mL/m^2^, and peak tricuspid regurgitation (TR) velocity >2.8 m/sec. However, diastolic function can be determined only when more than half of these parameters are concordant. Between 11% and 22% of scans are labeled indeterminate according to real-world clinical data [68,71,72].

The application of bidimensional STE, mainly used for the objective quantification of systolic myocardial deformation, has been recently studied also for the evaluation of diastolic function. New echo-parameters have been found to correlate well with LV filling pressure; this could help in the “grey-zone” when diastolic function is indeterminate. LV global longitudinal diastolic strain (Ds) rate (DSr) measurements during the isovolumic relaxation period and during early diastole by STE are the two variables that best correlate with the time constant of LV relaxation (t) [73]. Dokainish [74] showed that 2D–derived global Ds and DSr (during peak trans-mitral filling) correlated well with invasively measured LV diastolic function. Moreover, E/Ds was a better predictor of LV filling pressure than E/E’: global Ds may provide a more complete reflection of overall LV diastolic function than E’ velocity at the mitral annuli due to its angle-independency. These novel parameters have been found to predict outcomes in several conditions [75,76], but technical challenges and the variability in strain rate measurements due to the available software used for analysis have limited their ordinary application. The STE has been implemented also for the LA evaluation. Studies have shown that LA myocardial strain and strain rate play important role in estimating cavity pressure, showing an inverse correlation with pulmonary capillary wedge pressure (PCWP) [77,78], even in DCM [79]. A recently meta-analysis showed that a peak atrial longitudinal strain (PALS, measured at the end of reservoir phase) <19% is a very accurate parameter for estimating raised PCWP (above 15 mmHg) [80]. Such measurement is easy to obtain except for patients with marked LA enlargement and LA areas with echo dropout. PALS results to be particularly reduced in patients with idiopathic compared to ischemic DCM and closely associated with functional capacity during exercise [81].

More recently, assessment of LV torsion using 2D STE has been proposed as a novel marker of LV relaxation. LV untwisting, the rapid reversal of rotation during early diastole, represents the rapid release of LV restoring forces and should be delayed and reduced in magnitude in patients with diastolic dysfunction. However, literature shows conflicting results regarding its usefulness for detecting diastolic dysfunction [82,83,84]. These conflicting results are probably related to the heterogeneity in populations and differences in cut-off used, so more studies are required [85].

Finally, a novel technique to evaluate diastolic function comes from AI. ML is a powerful tool able to process large datasets, like echocardiographic parameters of diastolic function, combined with detailed clinical and demographic features [86]. While E/A ratio has the limitation of a well-known U-shaped relationship with LV filling pressure, ML is particularly well-suited to detect and describe non-linear relationships [87], which is pertinent to diastolic assessment. ML has a proven incremental diagnostic value when compared to clinical diagnosis of HF with preserved EF [88,89]. This method was also applied to overcome limitations of current grading of diastolic disfunction. Lancaster et al. [90] found two clusters of grading, which proved this algorithm to be better in predicting mortality but not rehospitalizations. Even if ML can increase the accuracy of disease diagnosis and introduce a new classification of diastolic disfunction improving its prognostic ability, many limitations are still present. First of all, a large number and high-quality structured datasets are necessary to reflect real life practice [91]. Moreover, validation with independent external datasets for the generalization of the findings are still lacking [92].

Table 2 shows a comparison between Standard and Emerging echocardiographic techniques to assess diastolic function in dilated cardiomyopathy.

## 4. Right Ventricular Dilatation and Disfunction 

Right ventricle (RV) has been considered for many years the “forgotten chamber”. However, recent scientific literature highlighted the potential impact of right ventricular function on outcome of patients with DCM [93,94]. Assessing the RV function is difficult because of its complex anatomy and wall motion. Nowadays, CMR represents the gold standard for the evaluation of RV function and dimensions. However, CMR has limited availability and cannot be performed in specific patients (e.g., arrhythmic, claustrophobic and unstable patients). Therefore, echocardiography still plays a pivotal role in daily clinical-practice. The most used 2D echocardiographic parameters for the evaluation of RV function are tricuspid annular place systolic excursion (TAPSE), S’, fractional area change (FAC) and right ventricular index of myocardial performance (RIMP). TAPSE measured using M-mode directly measures the displacement of the lateral annulus of the tricuspid valve during the cardiac cycle. It is broadly used due to its facility, but it is influenced by the translational motion of the heart and by regional wall-motion abnormalities. Nevertheless, many authors have described its prognostic value in patients with DCM [95,96], even if inferior compared to FAC. Indeed, RV-FAC obtained by echocardiography has been shown to strongly correlate with CMR [97]. However, the evaluation of this latter parameter is challenging in case of suboptimal image quality. Another easy-echo parameter is tissue doppler imaging (TDI)-derived S’-wave velocity, which correlates well with other measures of global RV systolic function, but it’s main limitation is to be highly angle-dependent [98]. RIMP (also known as Tei index) is an index of global RV performance with undoubted prognostic value that can be calculated using either TDI velocity of the lateral tricuspid annulus or pulsed wave spectral Doppler ensuring that nonconsecutive beats have similar RR intervals [33,95].

A simple new parameter is the RV systolic to diastolic duration ratio (S/D ratio), assessed by continuous Doppler imaging using the TR envelope to calculate the durations of RV systole and diastole. Boqing Xu et al. [99] showed that an RV S/D ratio >1.2 was significantly associated with an increased one-year cardiac event rate in patients with advanced HF and DCM. However, a possible incremental prognostic value over clinical and routine echocardiographic parameters must be verified.

Strain imaging has been initially used to evaluate global LV function, but it is also able to provide a non-geometric approach to RV assessment. Recently 2D strain imaging using STE has been explored and RV global longitudinal strain (RVGLS) and global longitudinal strain rate (RVGLSR) have been shown to correlate well with RVEF measured by CMR [100]. RV strain derived from 2D STE detects subtle myocardial abnormalities and is only relatively angle- and load-independent [101]. STE could allow the reclassification from normal RV function (based on traditional parameters) to impaired RV function [102]. Moreover, RV strain has an important prognostic value in patients with HFrEF [103,104] and a better mortality prediction in HFrEF than conventional echocardiographic parameters, CMR-derived RVEF and CMR-derived RV strain [105]. Ishiwata et al. [106] found that FAC, TAPSE, RVLS (RV free-wall longitudinal strain) were independent predictors for primary outcome (composite of LV assist device implantation and all-cause death) and only the combined evaluation of FAC and RVGLS improved risk stratification in patients with DCM [107]. However, the current echocardiographic guidelines don’t recommend any definite reference range for either global or regional RV strain or strain rate due to the lack of validated data from large studies involving multivendor equipment [33].

The 2D echo techniques are only surrogates for the true RV volume, due to its crescent shaped. Instead, 3D echo evaluation is independent of the RV’s exact shape and does not rely on assumptions about the total structure. Indeed, 3D echo RV volumetric measurements correlate well with CMR measurements [108,109]. Vîjîiac et al. [110] showed that 3D RVEF <43.4% was the only independent predictor of adverse events in patients with DCM, compared with traditional 2D parameters.

Moreover, the recovery of RV function under therapy is frequent and can already be observed at 6 months. It precedes LVRR and is emerging as an early therapeutic target and an independent prognostic predictor [111]. Echocardiography has significantly improved in the last decade and, with the exponential technology advances, it may prove a primary modality to evaluate the RV in DCM, especially during the follow-up.

Table 3 shows a comparison between Standard and Emerging echocardiographic techniques to evaluate right ventricular disfunction in dilated cardiomyopathy.

## 5. Left Ventricular Dyssynchrony (LVD) and Left Ventricular Mechanical Dispersion 

LV contraction is the result of its coordinated mechanical activation following the propagation of the electrical impulse through the specialized conducting system. LVD can be defined as a significant delay of contraction between the different segments. It may or may not be secondary to electrical conduction tissue disturbance [112].

Asynchronous LV contraction has been hypothesized to play a key role in the likelihood of response to cardiac resynchronization therapy (CRT), even more important than QRS interval duration, especially in patients with DCM [113]. Comparison studies between echocardiographic techniques for estimating LVD and response to CRT have, however, returned conflicting results, to the point that the current guidelines still neglect this parameter in the decision-making algorithm for applying this therapy [114,115]. It is desirable that future studies address the identification of echocardiographic parameters not only capable of adequately measuring LVD but also of properly predicting outcomes after CRT. Many echocardiographic indices are used to evaluate LVD. The comparison of two opposite segments (generally the inferior septum and the lateral wall or the anterior septum and the posterior wall) and the evaluation of a single myocardial wall by M-mode echocardiography are the simplest [116,117].

TDI is one of the most used technique. Limitations of TDI are its angle-dependence, its being prone to noise and artifacts and the inability to distinguish between active and passive movements. When compared with more recent and technically complex methods such as STE longitudinal strain, TDI has showed better predictive value for chronic reverse remodeling in patients with LVD undergoing CRT [118]. The application of CMR tagging with longitudinal strain analysis of the LV myocardium showed how the correct evaluation of dyssynchrony requires the integration of the motion of the non-longitudinal (circumferential) fibers, which happens indirectly with the TDI evaluation [119]. Besides, information on LV dyssynchrony deriving from TDI proved to be comparable to that obtained with velocity-encoded cardiac CMR [120].

Apical transverse motion (ATM), a TDI-derived parameter defined as the apex motion perpendicular to the LV long-axis, quantifies the phenomenon of apical rocking as an integrative surrogate of both temporal and functional inhomogeneities within the LV. ATM has proven its superiority over conventional evaluation [121] and was closely associated with radial dyssynchrony measured by STE in a study of 35 patients with DCM [122].

Three-dimensional (3D) echocardiography, evaluating data on all the three basic components of LV function (longitudinal, radial and circumferential timing of all the myocardial segments), could accurately assess LVD. Nevertheless, 3D echocardiography suffers from the disadvantages associated with semi-automatic measurement and limited spatial resolution. Many studies have shown that 3D echocardiography is superior to TDI in assessing LV reverse remodeling and predicting response to CRT in DCM [123,124]. A combined approach of 2D and 3D techniques has shown incremental value for the prediction of LV reverse remodeling over use of only one technique [125].

STE has shown, through a more accurate analysis of radial dyssynchrony, a high sensitivity in identifying long-term responses to CRT [126]. It measures strain as active wall thickening which reflects true regional mechanics, whereas M-mode, TDI and 3D echocardiography volume displacement are affected by translation and tethering. STE, however, is dependent on frame rate, as well as on image resolution and noise and may oversimplify the complexity of LVD. A more advanced method, the 3D STE, merges the advantages of STE and 3D echocardiography and allows the coupling of the 3D area strain with both the 3D longitudinal and circumferential strain, which is more sensitive to changes in myocardial function. The main limitation is the relatively slow volume rate which may limit analysis of rapid events such as isovolumic contraction and relaxation phases [127].

A relatively recently analyzed echocardiographic parameter of mechanical dispersion as a possible marker of response to CRT is the systolic aortic root motion (SARM). It is a measure of the displacement of the aortic root during the cardiac cycle and is obtained through M-mode echocardiography. It has been shown to be able to predict non-response to CRT also in DCM patients [128].

Several attempts have been made to overcome the limitations inherent the methods previously described. One of them uses ML to combine different echocardiographic data, LVD parameters (ATM and SPE indices) and the duration of the QRS interval to try to predict the likelihood of LV reverse remodeling after CRT. It has been studied in 323 patients with heart failure (both ischemic and non-ischemic DCM) and it has been shown to improve the predicting value of standard imaging in the response to CRT [129]. Furthermore, AI has been used to characterize CRT responder profiles through clustering analysis, based on clinical and echocardiographic preimplantation data and quantitative analysis of longitudinal strain curves [130].

Table 4 shows a comparison between Standard and Emerging echocardiographic techniques to assess left ventricular dyssynchrony in dilated cardiomyopathy. 

## 6. Secondary Mitral and Tricuspid Regurgitation 

Secondary or functional MR develops in absence of structural anomalies (although minor leaflet thickening and annular calcification can be present) of the mitral valve. It is a disease of the atrium or the ventricle due to an imbalance between the tension acting on the mitral leaflets and the closing forces generated by the LV. Adverse LV remodeling leads to the apically displacement, tethering and restriction in mobility of the leaflets resulting in incomplete mitral valve closure [131,132]. In patients with DCM both leaflets exhibit a reduced systolic motion leading to incomplete coaptation.

Two-dimensional (2D) transthoracic echocardiography (TTE) and trans-esophageal echocardiography (TOE) supported by color-flow Doppler are the core methods used to analyze MR. The echocardiographic criteria to define the severity of MR only mildly differ between primary and secondary MR. Qualitative parameters include the presence of severe tenting and poor leaflet coaptation (these signs are specific, but their absence does not exclude severe regurgitation [133]), a large central jet (>50% of LA dimension) or an eccentric jet reaching the posterior wall [134] and a holosystolic dense or triangular continuous wave Doppler jet [135]. The measurement of the vena contracta, the narrowest portion of the regurgitant flow at or immediately below the regurgitant orifice, is a semiquantitative index and in secondary MR appears to be rather elongated along the mitral coaptation line and non-circular [136]. Other semiquantitative parameters are systolic flow reversal in the pulmonary veins, an E-wave dominant at the pulse-wave Doppler of the mitral inflow and the mitral to aortic time-velocity integral ratio >1.4 [137]. Effective regurgitant orifice area and regurgitant volume give additional information, with lower thresholds in secondary MR [138]. In the end, LV and/or LA dilatation argue in favor of the severity of the valve defect.

Three dimensional (3D) TTE and TOE allow a more precise prediction of regurgitation severity based on a comprehensive evaluation of valve morphology and leaflet coaptation, an accurate quantification of LV dimensions, a detailed geometry of vena contracta area and effective regurgitant orifice area and an automated quantitation of flow and regurgitant volume by 3D color-flow Doppler [139,140]. Three dimensional (3D) echocardiography has been recently applied to automatic quantify leaflet tethering by measuring the tethering distance from papillary muscle tip to the mitral annulus and measuring the tenting volume [141].

2D techniques were able to correctly quantified MR severity in less than two-thirds of cases while 3D technique showed higher accuracy in identifying severe MR compared to cardiac CMR [142]. Nevertheless, although the numerous improvements in recent years, the lower spatial and temporal resolution of 3D TTE affects its evaluation of valvular structures [133].

Secondary TR is the most common cause of tricuspid insufficiency caused by dilation of the RV and/or of the tricuspid annulus due to left-sided heart valve diseases, pulmonary hypertension, congenital heart defects, and cardiomyopathy [131]. Most Doppler methods used to assess MR are directly applicable to the quantification of TR. The main difference is that TR jet is usually a lower pressure and lower velocity than is typically found in MR [143]. Despite the volumetric estimation of right heart constitutes the principal challenge of 3D echocardiography, several studies proved its usefulness to define the severity of TR [144,145].

AI-based valve assessment software algorithms integrating 2D and 3D echocardiographic parameters with automated quantification of disease severity have shown to reduce time to analyze cardiac structures and provide good reproducibility with minimal user intervention. Their implementation could improve diagnostic and prognostic stratification for surgical and interventional procedures [146,147].

Table 5 shows a Comparison between Standard and Emerging echocardiographic techniques to evaluate mitrale and tricuspid regurgitation in dilated cardiomyopathy.

## 7. Left Ventricular Thrombus

Autopsy [148] and echocardiographic studies [149] have revealed that the incidence of left ventricular thrombus (LVT) in DCM is about 11–44% with a subsequent incidence of embolism around 11–20% [150]. LVT can remain underdiagnosed since 2D TTE misses two-thirds of thrombi, especially if these are small in size and located at the LV apex [151]. The advent of ultrasound contrast agents [152], providing the opacification within the cardiac chambers to demonstrate the avascular “filling defect” appearance of an intracardiac LVT, has improved the diagnostic accuracy of TTE from 82 to 92% [153]. Real-time 3D echo provides an unlimited number of cutting planes in all directions through a single full volume dataset that can be cropped and rotated improving sensitivity and reducing the risk of missing small apical thrombi [154]. However, this technique is not able to distinguish between LVT and myocardium or to evaluate the changes in thrombi structure, as the different shades of blue/brown color visualized by 3D echo reflect the depth of different structures rather than their texture [155]. Conversely, the myocardial deformation assessed by tissue Doppler imaging (TDI) using strain-rate (SR) techniques allows to differentiate between fresh (range: 5–27 days) and old (4–26 months) LVT [156]. Besides, as older thrombi are more collagen rich than fresh thrombi, they appear structurally better organized and stiffer and exhibit lower deformation values (expressed as lower peak SR) because of the changing intraventricular pressure during the isovolumetric contraction and relaxation periods [156]. Finally, nowadays AI technology is applied to recognize and classify intracardiac masses such as LA thrombosis, cardiac tumors and vegetation so it is realistic to expect AI to be a near-future application for LVT diagnosis [66,157]. Most of the aforementioned new echocardiographic technologies have only been tested in ischemic DCM, therefore it would seem appropriate that future studies will focus on their application in non-ischemic DCM patients.

Table 6 shows a comparison between Standard and Emerging echocardiographic techniques to identify left ventricular thrombus in dilated cardiomyopathy.

## 8. Myocardial Scars and Fibrosis 

In DCM patients, myocardial fibrosis (MF) is commonly found and contributes to the weakening and dilatation of the ventricular walls, affects the mechanical-electrical activity of cardiomyocyte and it is associated with deteriorating cardiac function and high long-term mortality [158,159]. The identification of scars and fibrosis burden is helpful for risk stratification and for determining the timing of early intervention [160]. The idea of using echocardiography to detect MF dates back to 1980s [161]. Since histopathologic and histochemical studies suggested that the increase in echo amplitude was correlated with myocardial scar formation an MF deposition, a landmark study proposed the calibrated myocardial integrated backscatter (IB) analysis as an echo technique able to identify MF [162]. Subsequently, Mizuno et al. first succeeded in evaluate quantitatively the severity of MF in DCM using combined tissue harmonic imaging (THI) and IB analysis [163]. Unfortunately this approach failed though to demonstrate feasibility in the real-life clinical practice [164].

Four new echo techniques, mostly studied in ischemic patients, are now available to identify and quantify MF and may be usefully applied in DCM:

1. Contrast-enhanced (CE) 3D Echo. Montant et al. tested CE-3D echo versus CMR-LGE and found that second-harmonic imaging (with transmission/receive 1.6/3.2 Mhz) at a mechanical index of 0.5 was the best combination to differentiate normal myocardium from fibrotic scar [165].
2. Three dimensional (3D) speckle tracking echocardiography. A study conducted in DCM patients prior to heart transplant comparing STE versus histological findings found that 3D GLS may be an optimal surrogate marker for reflecting MF (Area Under Curve, AUC 0.86) [166].
3. Pulse cancellation ultrasound technique (eSCAR). The echo machine built-in setting for LV opacification used without CE, thanks to cancellation of “linear” signals back from normal myocardium, is incidentally very efficient to enhance signals from abnormal myocardial tissue, such as fibrotic [167] and calcific tissues [168], which on the contrary show “nonlinear” response. This technique is able not only to provide a semi-quantitative identification of MF (number of scarred segments), but also to simply define a binary response “scar: yes/no” by the use of the software binary filter (“default” thresholding method) [167]. Furthermore, eScar has been shown to be able to identify scar burden as a prognostic marker for ICD appropriate discharge [169].
4. Radiomics-based texture analysis. Using a ML pipeline to integrate and process ultrasound images texture features the presence of MF is predictable with an AUC of 0.84 (sensitivity 86.4% and specificity 83.3%) [170].

Table 7 shows a comparison between Standard and Emerging echocardiographic techniques to assess myocardial scars and fibrosis in dilated cardiomyopathy.

## 9. Conclusions

Exponential growth of echocardiography’s technology and performance in recent years has not only resulted in improved diagnostic accuracy of DCM but highlighted the complexities of DCM characterization that extend beyond simplistic volumetric, flow and pressure determination. Although nowadays CMR remains the gold standard technique in DCM, the echocardiographic advances and novelties summarized, if properly integrated into clinical practice in the coming years, could bring echocardiography closer to CMR in terms of accuracy. Furthermore, unlike CMR, the use of affordable, accessible echocardiographic techniques applicable in unstable, arrhythmic and claustrophobic patients could prove in the future to be the new gold standard to track the longitudinal follow-up of patients affected by DCM. The application in DCM patients of the aforementioned echocardiographic advances must further be corroborated by large clinical studies, mapping out an interesting emergent research area for scholars in the near future.

## Figures and Tables

**Table 1:** Comparison between Standard and Emerging echocardiographic techniques to assess left ventricular dimensions, geometry and systolic function in dilated cardiomyopathy.

Assessing Left Ventricular Dimensions, Geometry and Systolic Function					
Standard Echocardiographic Techniques			Emerging Echocardiographic Techniques		
Technique Name and Related Parameters	Limitations	New Recent Findings	Technique Name and Related Parameters	Potential Benefits	Current Key Studies in DCM
**2D Transthoracic Echocardiography**	Conditioned by foreshortening of apex Based on geometrical assumptions; Risk of endocardial dropout; Conditioned by shape distortions.	Corrects for shape distortions; Less geometrical assumptions compared with linear dimensions [8].	**Non-invasive LV pressure-strain loop and GWI**	Powerful and independent predictor of outcome; Deepens the relationships between LV remodeling and increased after-load; Better predicts LV fibrosis; Useful to assess therapeutic response.	**Prospective Study** [36]
**3D Transthoracic Echocardiography**	Lower temporal resolution; Lacking data on normal values; Image-quality dependent.	No geometrical assumption Unaffected by foreshortening More accurate and reproducible compared to other imaging modalities Predictive of CRT response [32].	**Reverse remodeling index**	It is an independent predictor of all-cause mortality and heart transplantation	**Prospective Study** [44]
			**Post-systolic shortening and Early systolic lengthening**	It can predict adverse cardiac outcomes.	**Retrospective Study** [48]
**GLS**	Lacking set of normal values; High endor-dependent.	Angle independent High prognostic value Predict major arrhythmic events independently from EF [22].	**MVC tissue longitudinal elongation**	It can predict LV remodeling sphericity leading to new diagnostic tools.	**Retrospective Study** [57]
			**Artificial intelligence**	Reduce observer variability, providing more consistent and reproducible data; Allow big data analysis, predicting future data; Time and cost saving.	**Retrospective Study** [63]

DCM = dilated cardiomyopathy; PW = Pulsed wave; LV = left ventricle; 2D = Two dimensions; 3D = three dimensions; CRT = cardiac resynchronization therapy; EF = ejection fraction; GWI = global work index; MVC = mitral valve complex.

**Table 2:** Comparison between Standard and Emerging echocardiographic techniques to assess diastolic function in dilated cardiomyopathy.

Diastolic Function Assessment					
Standard Echocardiographic Techniques			Emerging Echocardiographic Techniques		
Technique Name and Related Parameters	Limitations	New Recent Findings	Technique Name and Related Parameters	Potential Benefits	Current Key Studies in DCM
PW Doppler(E/A ratio)	U-shaped relation with LV diastolic function; Preload dependent.	Easy to obtain and interpret in most cases; Strong predictor of mortality in DCM, independently from EF and age.	Ventricular 2D-Speckle Tracking (Ds, DSr)	Better predictor of LV filling pressure; SRe: predictor of response to therapy in DCM.	Prospective Study [77]
Tissue Doppler Imaging (E/E’)	Highly angle dependent; Presence of a grey zone.	Preload independent; Correlation with heart catheterization Tau; Applicable in several diseases [70].	Atrial 2D- Speckle Tracking	Easy to perform; possibility of off-line processing; Practical for serial follow-up; Angle independent; PALS associated with functional capacity during exercise in DCM.	Prospective Study [88]
2D echocardiography (LAVi)	Elevated volume index in several other conditions: AF, atrial flutter, mitral valve diseases, high-output states (e.g., anemia).	Efficiently reflects cumulative effects of LV filling pressure [68].	Artificial Intelligence and Machine Learning	Improves diagnostic accuracy, reducing indeterminate classification Enhances prognostication Opens to novel parameters.	Retrospective Study [88]

DCM = dilated cardiomyopathy; PW = Pulsed wave; LV = left ventricle; EF = ejection fraction; 2D = Two dimensions; AF = atrial fibrillation; Ds = diastolic strain; DSr = diastolic strain rate.

**Table 3:** Comparison between Standard and Emerging echocardiographic techniques to evaluate right ventricular disfunction in dilated cardiomyopathy.

Right Ventricular Disfunction Evaluation					
Standard Echocardiographic Techniques			Emerging Echocardiographic Techniques		
Technique Name and Related Parameters	Limitations	New Recent Findings	Technique Name and Related Parameters	Potential Benefits	Current Key Studies in DCM
**Fractional Area Change (FAC)**	Challenging in case of suboptimal image quality of RV free wall; Only acceptable inter-observer reproducibility; Neglects the contribution of RV outflow tract.	RV-FAC provide better prognostic information than TAPSE or S’ in DCM and has been shown to strongly correlate with CMR [97].	**RV S/D ratio at CW Doppler**	Easy to perform; Prognostic value in advanced HF with DCM.	**Prospective Study** [99]
**TAPSE**	Angle-dependent; Influenced by regional wall-motion abnormalities.	It is an accurate marker of RV dysfunction in pediatric patients with DCM [107].	**2D RV Speckle tracking**	Angle and load-independent; Good correlation with RVEF at CMR; Better mortality predictor than other echocardiographic or CMR-parameters.	**Prospective Study** [105] **Retrospective Study** [106]
**Tissue Doppler Imaging** **(S’)**	Angle-dependent; Not representative of RV global function after thoracotomy, pulmonary thromboendarterectomy or heart transplantation.	S’ combined with increased plasma BNP additively predict adverse cardiac outcomes in DCM [98].	**3D echocardiography**	Includes RV outflow tract; correlates well with EF by CMR; Independent prognostic value in DCM; Practical for serial follow-up.	**Prospective Study** [110]

DCM = dilated cardiomyopathy; TAPSE = tricuspid annular plane excursion; CW = Continuous wave; RV = right ventricle; 2D = Two dimensions; 3D = Three dimensions; EF = ejection; CMR = cardiac magnetic resonance.

**Table 4:** Comparison between Standard and Emerging echocardiographic techniques to assess left ventricular dyssynchrony in dilated cardiomyopathy.

Left Ventricular Dyssynchrony Assessment					
Standard Echocardiographic Techniques			Emerging Echocardiographic Techniques		
Technique Name and Related Parameters	Limitations	New Recent Findings	Technique Name and Related Parameters	Potential Benefits	Current Key Studies in DCM
**Standard 2D and M-Mode echocardiographic detection of LVD**	Lack of correlation with CMR; Modest specificity and sensitivity; Unable to analyse the various components of the cardiac contraction movement; Conditioned by translation and tethering effects.	Time differences between early septal and delayed displacement of posterolateral wall on M-mode images improve the predictive ability for CRT responses [117].	**Apical Trasverse Motion by Tissue Doppler Imaging**	Proved superior over conventional techniques to define LVD; Precise assessment of radial dyssynchrony.	**Cross-Sectional Study** [122]
			**3D Echocardiography**	Good ability in identifying mechanical delays in myocardial walls; Possibility of obtaining data on longitudinal, radial and circumferential timing of the myocardial segments.	**Prospective Study** [123]
**Tissue Doppler Imaging**	Extensively angle-dependent; Prone to noise and artifacts; Inability to distinguish between active and passive movements.	Good correlation with velocity-encoded cardiac CMR; Good predictive value for LVRR in patients undergoing CRT [118].	**3D GLS Speckle Tracking**	Reflect true regional mechanics Allows the coupling of 3D area strain with both 3D longitudinal and circumferential strain; More sensitive to changes in myocardial function.	**Prospective Study** [127]
			**Systolic Aortic Root Motion**	Easily obtained by M-Mode echocardiography; Good ability to predict non-response to CRT.	**Retrospective Study** [128]
**2D GLS Speckle Tracking**	Angle-dependent; Dependent on frame rate, image resolution and noise; Risk of oversimplifying the complexity of LVD.	High sensitivity in identifying long-term responses to CRT [126].	**AI technology**	Combines LVD echocardiographic, electrocardiographic and clinical parameters to improve the predicting value of imaging approaches for the response to CRT.	**Retrospective Study** [130]

DCM = dilated cardiomyopathy; LVD = left ventricular dyssynchrony; GLS = global longitudinal strain; CRT = cardiac resinchronization therapy; LVRR = left ventricle reverse remodeling; 2D = Two dimensions; 3D = Three dimensions; CMR = cardiac magnetic resonance.

**Table 5:** Comparison between Standard and Emerging echocardiographic techniques to evaluate mitrale and tricuspid regurgitation in dilated cardiomyopathy.

Secondary Mitral and Tricuspid Regurgitation Evaluation					
Standard Echocardiographic Techniques			Emerging Echocardiographic Techniques		
Technique Name and Related Parameters	Limitations	New Recent Findings	Technique Name and Related Parameters	Potential Benefits	Current Key Studies in DCM
**Standard 2D Transthoracic and Trans-oesophageal Echocardiography with Color Doppler**	Highly influenced by settings, hemodynamic conditions, dynamic changes in the orifice area and mechanism of mitral and tricuspid regurgitation; Need for cumbersome manual measurements in which small errors result in significant inaccuracies; Risk of overestimation of valvular defects; Corrected quantification of the severity in less than two-thirds of cases.	For proximal flow convergence: Rapid qualitative assessment; Absence of PISA is usually a sign of mild regurgitation; For VC; Surrogate for regurgitant orifice size; Independent of flow rate; Can be applied to eccentric jets; Less dependent on technical factors; >For jet area; Easy to measure [132].	**Real-Time 3D Transthoracic and Trans-oesophageal Echocardiography with color Doppler**	Faithful reconstruction of the valve anatomy; More accurate measurements of quantitative and semiquantitative parameters; Good reproducibility with CMR findings Availability of automated approaches more accurate and reproducible than spectral Doppler velocity profiles and 2D areas; Higher accuracy to identify severe regurgitation than CMR.	**Metanalysis:** [142]
			**AI technology**	Integration of 2D and 3D echocardiographic parameters with automated quantification of disease severity; Reduced time to analyze cardiac structures and good reproducibility with minimal user intervention.	**Review** [146]

DCM = dilated cardiomyopathy; 2D = Two dimensions; 3D = Three dimensions; PISA = Proximal Isovelocity Surface Area; VC = vena contracta; CMR = cardiac magnetic resonance; AI = artificial intelligence.

**Table 6:** Comparison between Standard and Emerging echocardiographic techniques to identify left ventricular thrombus in dilated cardiomyopathy.

Identifying Left Ventricular Thrombus					
Standard Echocardiographic Techniques			Emerging Echocardiographic Techniques		
Technique Name and Related Parameters	Limitations	New Recent Findings	Technique Name and Related Parameters	Potential Benefits	Current Key Studies in DCM
**Standard 2D Transthoracic Echocardiography**	Misses up to two-thirds of thrombi → very low sensitivity.	The majority (56%) of LVT in DCM are diagnosed by Standard Transthoracic Echocardiography [155].	**Real-Time 3D Echocardiography**	Identification of the attachment point of the thrombus to the cardiac wall; Delineation of the changes in thrombi structure (e.g., calcification, degeneration); More accurate assessment of thrombus mobility; Accurate calculation of thrombus volume.	**Cross-Sectional** [154]
**Contrast-Enhanced 2D Echocardiography**	Need for ultrasound contrast agents; Time consuming; Not able to evaluate the changes in thrombi structure.	Contrast-Enhanced echocardiography o nearly doubled sensitivity and yielded improved accuracy versus non-contrast echo [152]	**Strain-Rate by Tissue Doppler Imaging**	Allows to differentiate between fresh (range: 5–27 days) and old (4–26 months) thrombi; Allows to obtain an approximate calculation of the thrombus stiffness.	**Missing specific data in non-ischemic DCM → studies are needed**
			**AI technology**	Already applied to recognize intracardiac masses (e.g., left atrium thrombosis, cardiac tumors and vegetation) → it is realistic to expect AI to be a near-future application for detect LVT	**Missing specific data in non-ischemic DCM → studies are needed**

DCM = dilated cardiomyopathy; LVT = left ventricular thrombus; 2D = Two dimensions; 3D = Three dimensions; CMR = cardiac magnetic resonance; AI = artificial intelligence.

**Table 7:** Comparison between Standard and Emerging echocardiographic techniques to assess myocardial scars and fibrosis in dilated cardiomyopathy.

Assessing Myocardial Scars and Fibrosis					
Standard Echocardiographic Techniques			Emerging Echocardiographic Techniques		
Technique Name and Related Parameters	Limitations	New Recent Findings	Technique Name and Related Parameters	Potential Benefits	Current Key Studies in DCM
**THI-Calibrated Myocardial Integrated Backscatter** **2D Echocardiography**	Lack of correlation with CMR; Lack of correlation with histopathology findings; Failed though to demonstrate feasibility in the real-life clinical practice.	It is the only echocardiography technique proven to evaluate quantitatively myocardial fibrosis specifically in DCM patients [163].	**Contrast-Enhanced 3D Echocardiography**	Strong correlation with CMR; Available and cheaper than CMR; Rapid learning curve.	**Missing specific data in non-ischemic DCM → studies are needed**
			**3D GLS Speckle Tracking**	Strong correlation with histopathology findings; Good intra/inter-observer reproducibility; Practical for serial follow-up.	**Cross-Sectional Study** [166]
			**Pulse Cancellation Ultrasound** **[eSCAR]**	Standard 2D phase array probe with contrast opacification preset (power-modulation/pulse inversion harmonic imaging; transmit 1.6 MHz/receive 3.2 MHz) without the need for contrast administration; Strong correlation with CMR Prognostic value for ICD appropriate discharge; Bedside, fast and easy, perfect for screening.	**Missing specific data in non-ischemic DCM → studies are needed**
			**Radiomics-Based Texture Analysis**	Simple and cheap application of AI to standard echocardiography software; Strong correlation with CMR Vendor-independent; Good interobserver agreement.	**Cross-Sectional Study** [170]

DCM = dilated cardiomyopathy; THI = tissue harmonic imaging; 2D = Two dimensions; 3D = Three dimensions; CMR = cardiac magnetic resonance; GLS = global longitudinal strain; AI = artificial intelligence.

## Data Availability

Not applicable.
